# Numerical analysis of rectangular type batch ohmic heater to identify the cold point

**DOI:** 10.1002/fsn3.1353

**Published:** 2019-12-20

**Authors:** Won Choi, Sang‐Soon Kim, Sang‐Hyun Park, Jun‐Bae Ahn, Dong‐Hyun Kang

**Affiliations:** ^1^ Department of Landscape Architecture and Rural Systems Engineering, and Research Institute for Agriculture and Life Sciences Seoul National University Seoul Republic of Korea; ^2^ Department of Food Engineering Dankook University Cheonan Chungnam Republic of Korea; ^3^ Department of Food Science and Technology Kongju National University Yesan Chungnam Republic of Korea; ^4^ School of Food Service & Culinary Arts Seowon University Cheongju Chungbuk Republic of Korea; ^5^ Department of Agricultural Biotechnology Center for Food and Bioconvergence, and Research Institute for Agricultural and Life Sciences Seoul National University Seoul Republic of Korea; ^6^ Institutes of Green Bio Science & Technology Seoul National University Pyeongchang‐gun Gangwon‐do Republic of Korea

**Keywords:** cold point, *E. coli* O157:H71, numerical simulation, Ohmic heater, orange juice

## Abstract

The objective of this research was to precisely simulate the temperature distribution and inactivation of *Escherichia coli* O157:H7 by batch ohmic heating pasteurization of orange juice based on a time‐dependent numerical model. A finite element method (FEM) embedded with pathogen inactivation codes using Java language simultaneously solved electric heating, k‐ε turbulent flow, and heat transfer equations and dealt with natural heat losses through the walls and air as the boundary conditions. The simulated temperature distribution and populations of *E. coli* O157:H7 did not differ from the experimental data for every treatment time within a relative error of 6.0%. A cold point problem was observed in the bottom corner, which was more severe for large orange juice samples, leading to an increased treatment time in order to ensure a 5‐log reduction of *E. coli* O157:H7.

## Graphical abstract

- Two‐step process by ohmic heating was successfully simulated by numerical approach.
- Cold point, of which pathogen can survive, was found at the edges of the treatment chamber.
- The mathematical method can be used to adjust processing time considering sample volume.

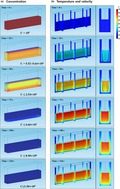

## 1 INTRODUCTION

Fruit juices enjoy worldwide popularity and have been considered microbiologically safe foods due to their acidity (Sospedra, Rubert, Soriano, & Manes, 2012). However, many research investigations indicate that foodborne pathogens and food spoilage bacteria can survive in fruit juice by means of acid‐resistant responses (Castillo, Villarruel‐López, Navarro‐Hidalgo, Martínez‐González, & Torres‐Vitela, 2006; Enache & Chen, 2007; Oyarzabal, Nogueira, & Gombas, 2003). For example, outbreaks of *Salmonella* Hartford and *Escherichia coli* O157:H7 infections associated with orange juice and apple juice, respectively, have been reported despite of their acidity (Besser et al., 1993; Cody et al., 1999; Cook et al., 1998). These microorganisms can be inactivated easily at high temperature (>80°C), but food quality values, such as color and nutritional value, could be damaged by high temperature. Therefore, several alternative technologies, including thermal and nonthermal treatments, have been applied to pasteurize fruit juices while inducing minimal quality degradation (Ait‐Ouazzou, Espina, García‐Gonzalo, & Pagán, 2013; Silva, Tan, & Farid, 2012; Timmermans et al., 2014). Advanced heating technologies such as microwave heating, radio frequency heating, infrared heating, and ohmic heating have the advantages of volumetric, uniform, and rapid heating. Ohmic heating can be effectively used in the food industry to provide microbiologically safe, high‐quality foods. Inactivation of foodborne pathogens, food spoilage bacteria, and enzymes in fruit juice by ohmic heating has been of interest as an alternative technology to conventional heating recently (İçi, Yildiz, & Baysal, 2008), and many research investigations related to the pasteurization of liquid and liquid–solid food products by ohmic heating have been reported (Choi, Nguyen, Lee, & Jun, 2011; Leizerson & Shimoni, 2005a). Ohmic heating has been verified as one effective method to inactivate *E. coli* O157:H7, *S*. Typhimurium, *Listeria monocytogenes*, and *Alicyclobacillus acidoterrestris* spores in orange juice (Baysal & İçier, 2010; Lee, Sagong, Ryu, & Kang, 2012), and the quality of ohmic‐heated orange juice was better than that of conventionally heated, infrared‐heated, or microwave‐heated orange juices (Leizerson & Shimoni, 2005b; Vikram, Ramesh, & Prapulla, 2005).

Rapid and uniform heating are the most important factors for improving food quality as well as ensuring microbiological safety by ohmic heating. Even though ohmic heating has been known as an advanced technology ensuring relatively uniform heating of foods, the heating uniformity is suboptimal. Several research investigations have reported that a cold zone can be observed in ohmic‐heated solid–liquid mixtures (Sarang, Sastry, Gaines, Yang, & Dunne, 2007) or at the junction of electrodes (Marra, 2014). This cold zone would not only serve as a reservoir of harmful microorganisms but also result in quality degradation of food due to over and under processing. In this regard, the identification of the exact temperature distribution of ohmic‐heated food is very important in food processing. Various methods have been utilized to identify the exact temperature distribution of ohmic‐heated samples. In the late 20th century, a two‐dimensional (2D) simulation was introduced to predict the temperature distribution in a static ohmic heating chamber (Fu & Hsieh, 1999). After that, a three‐dimensional (3D) simulation was used to optimize ohmic heating processes (Knoerzer, Regier, & Schubert, 2006). Magnetic resonance imaging (MRI) temperature mapping was also used to observe the temperature distribution of static ohmic heating (Ye, Ruan, Chen, & Doona, 2004). Even though an MRI system can be used to observe the temperature in real time, the expense to establish the system is relatively high, and an additional location is needed. On the other hand, computational simulation can be used effectively to predict temperature distribution precisely without additional cost or system relocation (Shim, Lee, & Jun, 2010). Moreover, analysis performance improved dramatically with the development of the computer industry. Therefore, computational simulation was used in the present study to analyze the temperature distribution of ohmic heating.

Enzyme inactivation is a primary target of juice pasteurization, and there have been several research studies to identify the enzyme inactivation efficacy of ohmic heating (İçi et al., 2008; Leizerson & Shimoni, 2005a). However, inactivation of pathogens is another important objective of juice pasteurization, which consumers are interested in. Accordingly, we focused on pathogen inactivation in the present study. In South Korea, a few juice processing companies use small batch‐type ohmic heating systems without stirring equipment to produce a product of uniform temperature. This setup requires an excessive heating time to inactivate foodborne pathogens and food spoilage bacteria, which might also decrease food quality. Research investigations related to exact identification of the cold point in ohmic heating are limited. Thus, a three‐dimensional numerical model was developed to precisely estimate the temperature distribution and inactivation of *E. coli* O157:H7 in a static ohmic chamber without stirring equipment.

## 2 MATERIALS AND METHODS

### 2.1 Bacterial cultures and cell suspensions

*E. coli* O157:H7 (ATCC 43890, ATCC 35150, and ATCC 43889) was obtained from the bacterial culture collection of the School of Food Science, Seoul National University (Seoul, South Korea). A single colony cultivated from frozen stocks on tryptic soy agar (TSA, Difco) was inoculated into 5 ml of tryptic soy broth (TSB, Difco), incubated at 37°C for 24 hr, collected by centrifugation at 4,000 g for 20 min at 4°C, and washed three times with 0.2% peptone water (PW, Bacto). The final pellets were resuspended in 0.2% PW, corresponding to approximately 10^8–9^ CFU/ml. Afterward, suspended pellets of the three pathogen strains were combined to comprise a mixed‐strain cocktail containing approximately equal numbers of cells (10^7–8^ CFU/ml; counted by enumeration).

### 2.2 Sample preparation and inoculation

Pasteurized orange juice concentrate (66 °Brix), free of any preservatives, was purchased from a local grocery store (Seoul, South Korea). The soluble solid concentration of the orange juice was adjusted to 13 °Brix (pH 3.6) by mixing with distilled water to make reconstituted orange juice. Each sample was stored under refrigeration (4°C) and removed, 1 hr prior to inoculation and allowed to equilibrate to room temperature (27 ± 1°C). A mixed‐strain cocktail (0.2 ml) was inoculated into 30 ml orange juice samples before treatment.

### 2.3 Ohmic heater

**Figure 1:**
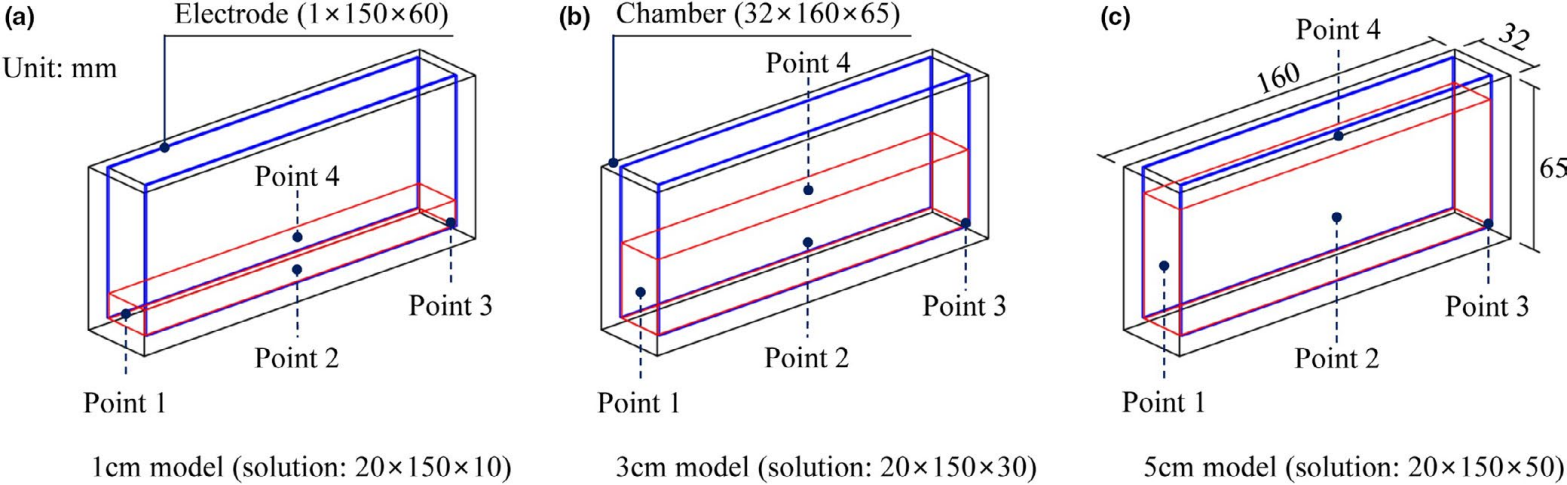
Points 1, 2, 3, and 4 of the ohmic chamber for 1, 3, and 5 cm models

The ohmic heating system (Kim & Kang, 2015) consisted of a function generator (catalog number 33210A, Agilent Technologies, Palo Alto, CA), a precision power amplifier (catalog number 4510, NF Corp., Yokohama, Japan), a data logger (catalog number 34970A, Agilent Technologies), two 0.1 × 15.0 × 4.0 cm titanium electrodes, and an ohmic heating chamber with inner dimensions of 2.2 × 15.0 × 6.0 cm and thickness of 0.5 cm (Figure 1). Two rectangular type titanium electrodes were installed in the ohmic heating chamber, and the distance between the two electrodes was 2.0 cm. Bottom sides of the electrodes were in contact with the juice, whereas upper parts were in contact with air. Thirty ml of sample equivalent to the 1.0 cm height was placed in the chamber, and an electric field of 25.6 V/cm with a pulsed waveform of 20 kHz and a duty cycle of 5% was applied to the electrodes. K‐type thermocouples were installed, and temperatures were recorded at 0.5‐s intervals with a data logger.

### 2.4 Microbial enumeration

For microbial enumeration, each treated 30 ml sample was immediately transferred into a sterile stomacher bag (Labplas Inc.) containing 270 ml of sterile 0.2% peptone water and homogenized for 2 min using a stomacher (Easy Mix, AES Chemunex). After homogenization, 1 ml of samples was 10‐fold serially diluted with 9 ml of sterile 0.2% peptone water, and 0.1 ml of diluents was spread‐plated onto Sorbitol MacConkey (SMAC, Difco) agar for enumeration of *E. coli* O157:H7. All plates were incubated at 37°C for 24 hr before counting colonies characteristic of the pathogens.

### 2.5 Material properties and model parameters

The material properties of orange juice are essential for simulating ohmic heating. Composition of orange juice by manufacturer was as follows: water (88.3%), protein (0.7%), fat (0.2%), carbohydrates (10.4%), and ash (0.4%). The following equations were utilized to predict the thermal conductivity (K, W/m·k), specific heat ($C_{p}$, J/kg·K), and density (ρ, kg/m^3^) of orange juice (Singh & Heldman, 2001) as follows.

(1) $$K=\sum_{i=1}^{n}K_{i}Y_{i}$$

(2) $$C_{p}=\sum_{i=1}^{n}C_{\mathrm{pi}}X_{i}$$

(3) $$\rho =\sum_{i=1}^{n}\rho_{i}X_{i}$$

where $K_{i}$, $C_{\mathrm{pi}}$, and $\rho_{i}$ are the thermal conductivity, specific heat, and density of the *i* th component, respectively; a food material has n components, *X_i_* is the weight fraction, and *Y_i_* is the volume fraction of the *i* th component, obtained as follows.

(4) $$Y_{i}=\frac{X_{i}/\rho_{i}}{\sum_{i=1}^{n}\left(X_{i}/\rho_{i}\right)}$$

Finally, the material properties were calculated as follows.

(5) $$K=-6.235\times 10^{-6}T^{2}+5.127\times 10^{-3}T-3.925\times 10^{-1}$$

(6) $$C_{p}=4.181\times 10^{-3}T^{2}-2.140T+4.144\times 10^{3}$$

(7) $$\rho =-3.318\times 10^{-4}T^{2}-1.461\times 10^{-2}T+1.053\times 10^{3}$$

where T is the absolute temperature (K).

#### 2.5.1 Dynamic viscosity

The dynamic viscosity (μ) of the orange juice is expressed as the function of the water activity ($a_{w}$) as follows (Ibarz, Gonzalez, & Esplugas, 1994).

(8) $$\mu =1.03\times 10^{-4}{\left(a_{w}\right)}^{-19.9}\exp\left(3,065/T\right)$$

where $a_{w}$ is 0.99 when the sugar content of the orange juice is 13 °Brix.

#### 2.5.2 Diffusivity

The diffusivity of water is linearly interpolated as follows.

(9) $$D_{w}=\left(0.0003\left(T-273.15\right)+0.2212\right)\times 10^{-6}\left(R^{2}=0.9732\right)$$

where $D_{w}$ is the diffusivity of water (m^2^/s).

The diffusivity (*D*) of various foods can be calculated as follows (Wilhelm, Suter, & Brusewitz, 2005).

(10) $$D=0.088\times 10^{-6}+\left(D_{w}-0.088\times 10^{-6}\right)X_{w}$$

where $X_{w}$ is the water mass fraction of the orange juice (0.883).

#### 2.5.3 Electrical conductivity

The electrical conductivity of orange juice is identified from voltage and current data (Palaniappan & Sastry, 1991) and calculated as follows.

(11) $$\sigma =\frac{\mathrm{LI}}{\mathrm{AV}}$$

where σ is the electrical conductivity (S/m), L is the distance between electrodes (m), I is the current (A), A is the cross‐sectional area of the electrodes to meet solution (m^2^), and V is the applied voltage (V).

The inner size of the ohmic chamber to measure electrical conductivity was empirically determined to be 2.2 × 2.0 × 2.5 cm with a thickness of 0.5 cm, and two 0.1 × 2.0 × 2.0 cm electrodes were installed inside the chamber. Orange juice equivalent to a height of 2.0 cm was placed in the chamber, and an electric field of 25.6 V/cm was applied to both electrodes to measure the voltage and current. The result showed a linear relationship depending on temperature as follows,

(12) $$\sigma =1.230\times 10^{-2}T-3.234\left(R^{2}=0.9962\right)$$

#### 2.5.4 D‐values related to pathogen inactivation

A constant‐temperature water bath (BW‐10G, Jeio Tech) was used for the determination of D‐values. Samples were equilibrated to target temperature by immersion in the constant‐temperature water bath. Pathogens were inoculated into 5 ml of orange juice to be 10^6^ CFU/ml in the tempered test tubes for D‐value experiments. A fiber‐optic temperature sensor (FOT‐L, FISO Technologies Inc.) connected to a signal conditioner (TMI‐4, FISO Technologies Inc.) was used to measure the temperature in the middle of the sample. The number of surviving pathogens was plotted on a logarithmic scale as a function of time (min). D‐value, the time needed to decrease the pathogen population by 90% (1 log), was calculated by the following equation.

(13) $$\log\left(N/N_{0}\right)=-t_{\min}/D_{t}$$

where $N_{0}$ is the initial population of the pathogen (CFU/ml), N is the population of the pathogen after treatment (CFU/ml), $D_{t}$ is the D‐value (min), and $t_{\min}$ is the treatment time (min).

Calculated D‐values were 14.90, 8.60, 1.55, and 0.26 for 45, 50, 55, and 60°C, respectively.

### 2.6 Mathematical model

Simulation for ohmic heating was conducted using COMSOL software (COMSOL 4.3, COMSOL Inc.) based on the FEM.

#### 2.6.1 Governing equation for electric field

Laplace's equation defining the electric field distribution in an ohmic conductor was given as follows.

(14) ∇·(σ(T)∇V)=0

The boundary and initial conditions for ohmic heating are given as follows.

(15) Electrode with a ground:V=0

(16) $$\text{Electrode with an electric potential}:V=V_{0}$$

(17) $$\text{Electrical insulation at the walls}:n\cdot \left(\sigma (T)\nabla V\right)=0$$

where n is the unit vector perpendicular to the boundary.

#### 2.6.2 Governing equation for heat balance

The governing equation including heat generation and transfer in flow condition is defined as follows:

(18) $$\rho C_{p}\frac{\partial T}{\partial t}=\nabla \cdot \left(K\nabla T\right)+q-\rho C_{p}u\cdot \nabla T$$

where *t* is the time (s), u is the velocity vector (m/s), and *q* is the heat source (W/m^3^) generated by the ohmic heater. The heat source can be calculated by the following equation.

(19) $$q=\sigma \left(T\right){\left|\nabla V\right|}^{2}$$

The heat flux on the boundaries with external natural convection cooling with air is given as follows.

(20) $$n\cdot \left(k\nabla T\right)=h\left(T_{\mathrm{ext}}-T\right)$$

where h is the heat transfer coefficient (W/m^2^∙K), and $T_{\mathrm{ext}}$ is the external temperature (K).

The heat transfer coefficient for external natural convection cooling can be changed depending on the geometrical configuration (vertical or horizontal face) of the heat transfer interface. The coefficient is also the function of the length (area/perimeter, m), internal and external temperatures, thermal conductivity, and so on. For most engineering purposes, the COMSOL software provides built‐in functions for the heat transfer coefficient, and thus, the heat transfer coefficient was automatically calculated by the software. The initial temperature was assumed to be 27°C.

#### 2.6.3 Governing equation for incompressible turbulent flow

The Rayleigh number (Ra) is an important dimensionless quantity used to classify internal fluid flow produced by free natural convection inside a chamber by laminar or turbulence (Equation 21).

(21) $$Ra=\frac{\alpha g\Delta TH^{3}}{\upsilon D}$$

where α is the thermal expansion coefficient (/°C), g is the gravitational acceleration constant (9.81 m/s^2^), υ is the kinematic viscosity (m^2^/s), and ΔT(K) and H (m) are the temperature difference and distance between warm and cold layers, respectively.

Rincón‐Casado, Sánchez de la Flor, Chacón Vera, & Sánchez Ramos (2017) defined that the Ra number to divide the laminar and turbulent regimes is 10^6^ in considering the natural convection heat transfers in the enclosures. Because the Ra values calculated approximately in our case were around 10^6^ to 10^8^, from an engineering point of view, the turbulent flow model was selected for fluid dynamics simulation. If the flow condition is in an incompressible state, the governing equations expressed as Cartesian coordinates can be described by the k‐ε turbulent flow model based on the Reynolds‐Averaged Navier‐Stokes (RANS) equations as follows.

(22) ∇·u=0

(23) $$\frac{\partial u}{\partial t}+\left(u\cdot \nabla \right)u=-\frac{1}{\rho }\nabla \cdot pI+\nabla \cdot \left[\left(\nu +\nu_{T}\right)\left(\nabla u+{\left(\nabla u\right)}^{T}\right)-\frac{2}{3}kI\right]+\frac{F}{\rho }$$

where p is the pressure (Pa), ν is the kinematic viscosity (m^2^/s), $\nu_{T}$ is the turbulent kinematic viscosity (m^2^/s), k is the turbulent kinetic energy (m^2^/s^2^), I is the identity vector, and F is the volumetric buoyancy force (N/m^3^).

Turbulent kinematic viscosity is associated with two dependent variables: turbulent kinetic energy and turbulent dissipation rate as follows.

(24) $$\nu_{T}=C_{\mu }\frac{k^{2}}{\varepsilon }$$

where $C_{\mu }$ is the constant model parameter, and ε is the turbulent dissipation rate (m^2^/s^3^).

The two parameters (k and ε) related to turbulent flow were calculated from two additional transport equations as follows.

(25) $$\frac{\partial k}{\partial t}+\left(u\cdot \nabla \right)k=\nabla \cdot \left[\left(\nu +\frac{\nu_{T}}{\sigma_{k}}\right)\nabla k\right]+p_{k}-\varepsilon$$

(26) $$\frac{\partial \varepsilon }{\partial t}+\left(u\cdot \nabla \right)\varepsilon =\nabla \cdot \left[\left(\nu +\frac{\nu_{T}}{\sigma_{\varepsilon }}\right)\nabla \varepsilon \right]+\frac{\varepsilon }{k}\left(C_{\varepsilon 1}p_{k}-C_{\varepsilon 2}\varepsilon \right)$$

where $p_{k}$ is the production term ($\nu_{T}\left[\nabla u:\left(\nabla u+{\left(\nabla u\right)}^{T}\right)\right]$) and $\sigma_{k}$, $\sigma_{\varepsilon }$, $C_{\varepsilon 1}$, $C_{\varepsilon 2}$ are the model parameters.

#### 2.6.4 Turbulent flow initial conditions

In the beginning of the simulation, the admissible mixing lengths were set up to be 0.07 at each domain to calculate the initial turbulent kinetic energies. After that, the turbulent kinetic energies were used to compute the initial turbulent dissipation rates. The initial values of velocities and pressures were set at zero.

(27) u=0

(28) p=0

(29) $$k_{\mathrm{init}}={\left(\frac{10\cdot \mu }{\rho \left(0.1\cdot l_{\mathrm{mix}}^{\lim}\right)}\right)}^{2}$$

(30) $$\varepsilon_{\mathrm{init}}=\frac{C_{\mu }k_{\mathrm{init}}^{3/2}}{0.1\cdot l_{\mathrm{mix}}^{\lim}}$$

where $l_{\mathrm{mix}}^{\lim}$ is the admissible mixing length (m), $k_{\mathrm{init}}$ is the initial turbulent kinetic energy (m^2^/s^2^), and $\varepsilon_{\mathrm{init}}$ is the initial turbulent dissipation rate (m^2^/s^3^).

#### 2.6.5 Turbulent flow wall conditions

The gradients of velocities and turbulent kinetic energies were in a state of homogeneous Neumann conditions, and the wall boundary conditions at the thin region near the walls in the flow variables with high gradients were defined by the wall functions as follows.

(31) u·n=0

(32) ∇k·n=0

(33) $$\left[\left(\nu +\nu_{T}\right)\left(\nabla u+{\left(\nabla u\right)}^{T}\right)-\frac{2}{3}kI\right]n=-\frac{u_{\tau }}{\delta w_{+}}u_{\mathrm{tang}}$$

(34) $$u_{\mathrm{tang}}=u-\left(u\cdot n\right)n$$

(35) $$\varepsilon =\frac{C_{\mu }k^{2}}{k_{v}\delta w_{+}\nu }$$

where $u_{\tau }$ is the friction velocity (11.06 m/s), $\delta w_{+}$ is the wall distance automatically calculated depending on the mesh size near the wall, $u_{\mathrm{tang}}$ is the tangential velocity (m/s), and $k_{v}$ is the model parameter (0.41).

#### 2.6.6 Governing equation for pathogen inactivation

The governing equation for pathogen inactivation was calculated as follows.

(36) $$\frac{\partial c}{\partial t}+\nabla \cdot \left(-D\nabla c\right)=R$$

where *c* is the concentration (CFU/ml·m^3^), and *R* is the reaction rate (CFU/ml·m^3^∙s).

Inactivation of *E. coli* O157:H7 is known to follow a first‐order reaction.

(37) R=-kc

The reaction rate constant (k, 1/s) to predict the inactivation of *E. coli* O157:H7 was defined as follows:

(38) $$k=\frac{2.303}{D_{t}}$$

For the boundary condition, there were no pathogen fluxes on the wall as shown by the following equation:

(39) u·(D∇c)=0

#### 2.6.7 Simulation setup

The temperature distribution of orange juice and inactivation of *E. coli* O157:H7 during ohmic heating were analyzed using COMSOL software. Our own pathogen inactivation algorithm coded by Java language was specially embedded into the software. AutoCAD (AutoCAD 2010, Autodesk, Inc.) software was used to create the 3D geometry of the ohmic chamber. The geometry was then imported into the COMSOL software. To increase convergence rate, the domains were discretized by free tetrahedral meshes using the advanced front method (AFM). The mesh sizes for each subdomain were manually controlled, and the averaged mesh quality (Q), defined as follows, was kept at 0.7576 throughout the whole domains.

(40) $$Q=\frac{4\sqrt{3}A}{h_{1}^{2}+h_{2}^{2}+h_{3}^{2}}$$

where A is the area of triangle, and $h_{1}$, $h_{2}$ and $h_{3}$ are the side lengths of the triangle.

**Figure 2:**
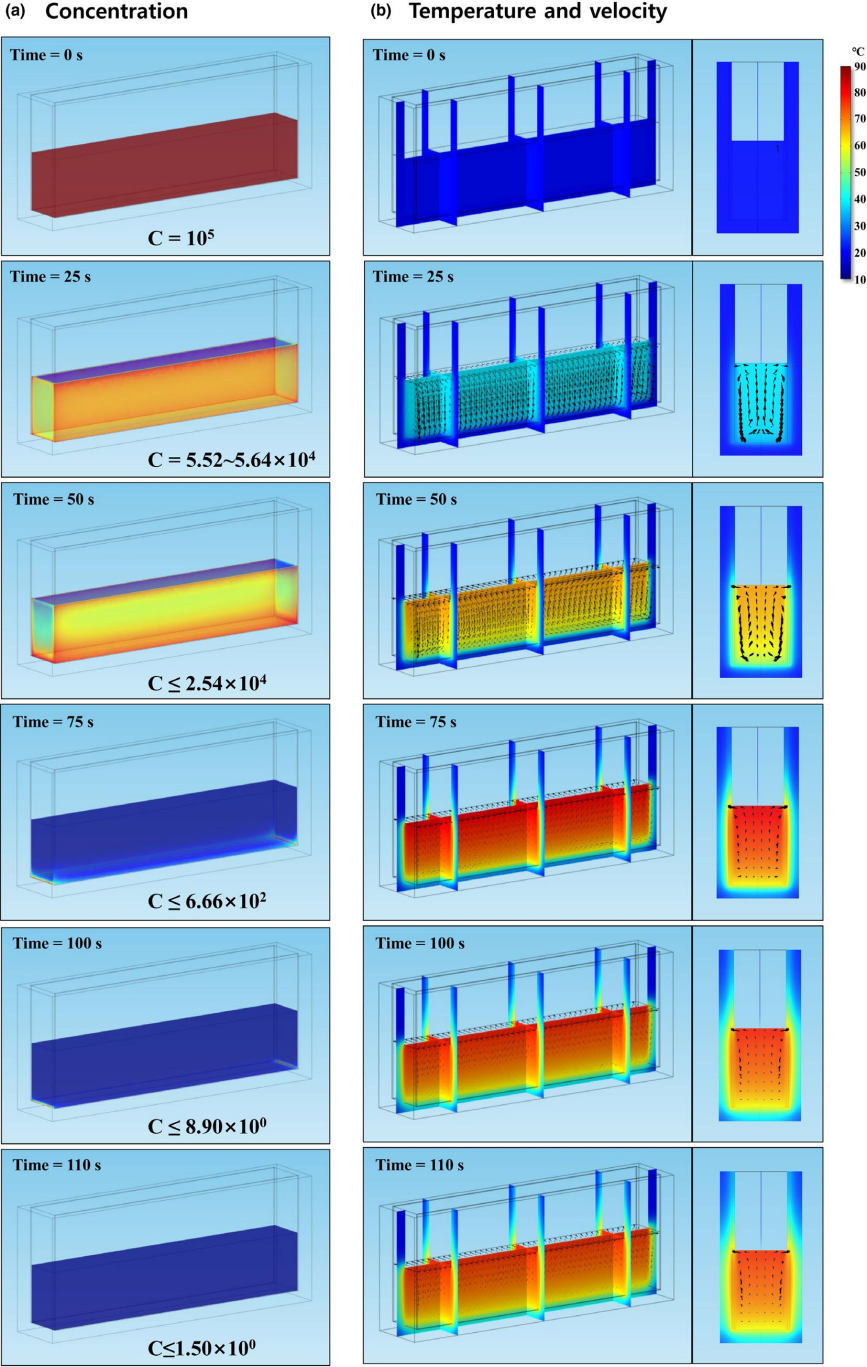
Simulation results for pathogen concentration, temperature distribution, and velocity distribution over treatment time (3 cm model)

**Figure 3:**
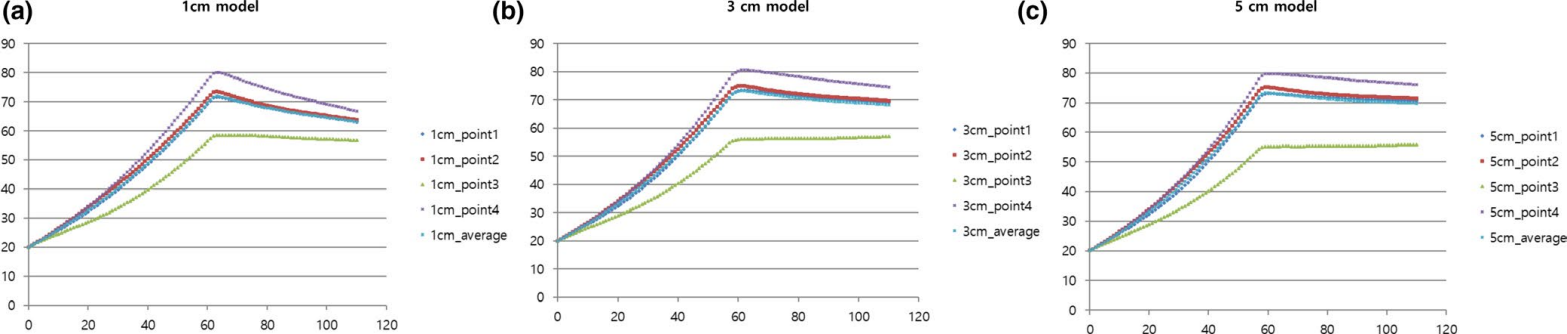
Simulation results of temperatures in models of various heights

The computational domain was finally discretized into 981,561, 801,721, and 620,960 tetrahedral mesh elements for the chambers with sample heights of 1, 3, and 5 cm, respectively (Figures 2 and 3). The partial differential equations (PDEs) including the heat balance, incompressible turbulent flow, pathogen inactivation, and electric field were simultaneously solved by a conjugate gradient (CG) iteration method coupled with a multifrontal massively parallel sparse direct solver (MUMPS), and a generalized minimal residual (GMRES) method coupled with parallel direct sparse solver (PARDISO), respectively. The transient simulation was executed with a time step of 0.1s and absolute tolerance of 0.010. In order to finish the simulation on a server‐level personal computer (Intel® Xeon® CPU X5690@4.00GHz (2 Processors), G.SKILL RAM 48GB@1,600MHz), approximately 0.7, 16.8, and 10.1 hr were required for sample heights of 1, 3, and 5 cm, respectively. It was observed that buoyancy forces generated by the ohmic heater caused stream flows in orange juice. To identify the heating rate in detail, we chose the following 4 points for investigating varying sample sizes of orange juice (Figure 1): side middle (point 1), center middle (point 2), bottom corner (point 3), and top middle (point 4), which were all located 1 mm from the boundaries. The temperatures, velocity distributions, and concentrations of *E. coli* O157:H7 were analyzed in the 1, 3, and 5 cm models for each treatment time during two‐step processing. The initial temperature and concentration of *E. coli* O157:H7 for simulating factory conditions determined to be 20°C and 10^5^ CFU/ml, respectively.

### 2.7 Statistical analysis

Experiments for investigating temperature increase and inactivation of *E. coli* O157:H7 were replicated three times. Orange juice temperatures and populations of *E. coli* O157:H7 were analyzed by *t* tests using Statistical Analysis System software (SAS 9.4, SAS Institute, Cary, NC). Significant differences between experimental and simulated results were determined at a significance level of *p* = .05.

## 3 RESULTS AND DISCUSSION

### 3.1 Model verification

Temperature distribution and inactivation of *E. coli* O157:H7 by ohmic heating were predicted by numerical simulation. The simulated data were compared with experimental results to validate the designed simulation model. Temperatures at the designated 3 points in the 1 cm model did not significantly differ (*p* > .05) between the simulated and experimental results (Table 1). The temperature increased most rapidly in the center middle (point 2) followed by the side middle (point 1) and the bottom corner (point 3) points. Because heat loss to the outside was a major contributor to heat transfer, the temperature at the center middle (point 2) was highest, whereas that at the bottom corner (point 3) was lowest (Figure 1). Ito, Fukuoka, & Hamada‐Sato (2014) also reported that temperature increase was most rapid at the center, and differences in heat transfer were pointed out as a reason. The simulated populations of *E. coli* O157:H7 were also not significantly different (*p* > .05) from populations enumerated on selective media for every treatment time interval (Table 2). Compared to previous studies, this study simulated ohmic heating, including the buoyancy forces that occurred in the solution, and accurately estimated the temperatures of solutions and populations of pathogens in real time within an error range of 6.0%. Because simulated results for temperature distribution and inactivation of *E. coli* O157:H7 were not significantly different from experimental results, we concluded that the designed computational simulation can accurately analyze the ohmic heating processing.

**Table 1:** Simulated and experimental temperatures of orange juice

Time(s)	Point 1 (°C)		Point 2 (°C)		Point 3 (°C)	
	Simulation	Experiment	Simulation	Experiment	Simulation	Experiment
0	27.01^a^	26.72 ± 1.25^a^	27.01^a^	26.78 ± 1.01^a^	27.01^a^	26.76 ± 1.07^a^
10	32.82^a^	31.66 ± 1.83^a^	33.89^a^	32.99 ± 0.76^a^	31.82^a^	30.81 ± 1.40^a^
20	38.80^a^	39.75 ± 2.57^a^	40.96^a^	42.05 ± 0.87^a^	36.32^a^	37.41 ± 2.09^a^
30	45.06^a^	46.24 ± 3.16^a^	48.33^a^	49.33 ± 0.61^a^	41.51^a^	42.88 ± 2.68^a^
40	51.61^a^	52.76 ± 3.21^a^	56.03^a^	56.34 ± 1.48^a^	47.64^a^	48.97 ± 2.68^a^
50	58.55^a^	61.45 ± 2.79^a^	64.25^a^	65.56 ± 3.00^a^	54.54^a^	57.39 ± 2.60^a^
60	66.02^a^	67.89 ± 2.42^a^	73.11^a^	72.12 ± 3.81^a^	62.07^a^	63.59 ± 2.46^a^

Note

Values in the same row for the same point followed by the same letter are not significantly different (*p* > .05).

**Table 2:** Simulated and experimental populations of *E. coli* O157:H7 in orange juice

Time (s)	Populations (log CFU/ml)	
	Simulation	Experiment
0	7.00^a^	6.93 ± 0.12^a^
20	6.80^a^	6.90 ± 0.21^a^
40	6.22^a^	6.23 ± 0.16^a^
50	5.37^a^	5.72 ± 0.41^a^
55	4.40^a^	4.14 ± 0.13^a^
60	3.05^a^	3.23 ± 0.24^a^

Note

Values in the same row followed by the same letter are not significantly different (*p* > .05).

### 3.2 Two‐step processing

Excessive heat treatment can cause quality degradation of food samples. For example, (Leizerson & Shimoni, 2005a) reported that vitamin C concentration decreased 7%–25% compared to that in fresh orange juice when the set temperature for ohmic heating exceeded 90°C. Therefore, we executed two‐step processing in which the first stage included continually applied ohmic heating until the top middle temperature of the orange juice reached 80°C, and in the second stage, electrical power was shut down to allow gradual inactivation of pathogens by latent heat.

#### 3.2.1 Temperatures

Orange juice temperature increased rapidly with ohmic heating treatment (Figures 2 and 3). After 60 s, the upper part of samples mostly showed temperature distributions of 70–80°C. On the other hand, the bottom corner of the samples reached temperatures of approximately 60°C. The center concentration effect was more pronounced in the higher height model because the heat losses at the center of the higher height model must be less than those of the low height model due to greater sample mass. The time intervals for the hottest point of each sample to reach 80°C were 62, 60, and 58 s, and the temperatures at the coldest points were 58.6, 56.8, and 55.2°C for the 1, 3, and 5 cm models, respectively (Figure 3). After shutting off ohmic heating, all temperatures except for point 3 (coldest region) cooled more rapidly in the smaller sample (1 cm model) than in the larger samples (3 or 5 cm models) and showed similar trends for average temperatures. On the other hand, the temperatures at the corners (point 3) of each sample did not change significantly (<2°C) after shutting down the ohmic heating, regardless of the sample height, because conduction heat transfers from solution to solid (chamber) were more active at the lateral and bottom walls rather than at the corner (Figure 3). The lowest temperatures after 110 s, treatment time to achieve 5 log reductions of *E. coli* O157:H7 were 56.8, 57.0, and 55.9°C for 1, 3, and 5 cm models, respectively, which indicates that more than 55°C (at the lowest point) is needed to ensure microbiological safety in juice products.

#### 3.2.2 Velocities

Many stream arrows were observed after 25 s treatment time, meaning that the convection effect inside the solution was actively progressed at this time (Figure 2). After that, hot fluids moving to both laterals cooled, became denser, and then sank to the bottom. Finally, this circulation pattern repeated itself, and rapid velocities were observed at the laterals of the chamber during the heating time. Based on these results, it is presumed that upward‐moving streamlines in most of the middle areas were first formed because hot fluids with lower densities rose due to buoyancy. Conversely, the directions of streamlines at the shallow lateral areas were downward, and their velocities would be greater than the upward‐moving streamlines following the theory of the continuum equation. Before the simulation, we set up the turbulent instead of the laminar equation because the Ra number calculated approximately was near the laminar‐turbulent transition zone. However, the simulation results consequentially showed that all flows observed inside the chamber were in a laminar state, and the flow velocities were under 0.001 m/s, except for the lateral areas. After shutting off the electric power, flow velocities slowed dramatically, and the mixing effect of juice sample due to convection currents inside the chamber became weak compared to heating time. The hottest temperatures were still observed at the top areas due to the buoyancy phenomenon, and the flow velocities at the top‐lateral areas were somewhat faster than other areas because the top areas were in direct contact with air.

#### 3.2.3 Pathogen concentrations

**Figure 4:**
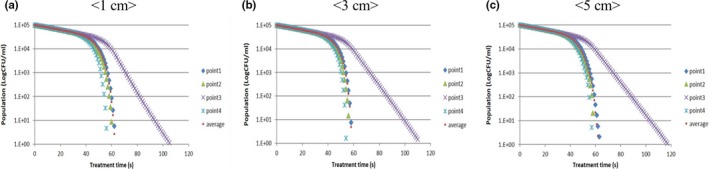
Simulation results of pathogen inactivation in models of various heights

The concentrations of *E. coli* O157:H7 decreased rapidly with ohmic heating treatment by means of volumetric resistive heating (Figure 4). Even though most *E. coli* O157:H7 were inactivated after 65‐s treatment time, some *E. coli* O157:H7 still survived at the bottom corner of the treatment chamber (point 3). Because as few as 10 organisms of *E. coli* O157:H7 can cause serious foodborne illness, these few surviving pathogens should not be overlooked (Radke & Alocilja, 2005). The U.S. FDA recommends following the 5‐log reduction performance standard, which means that juice processors should treat their juice using a process such as heat or UV‐C irradiation to achieve a 5‐log reduction in the number of target microorganisms (U.S. FDA., 2001). Therefore, it was necessary to identify how much time is needed to achieve 5‐log reduction of *E. coli* O157:H7 at various locations and model heights. Because temperatures increased most rapidly at point 4, *E. coli* O157:H7 was inactivated most rapidly at that locus regardless of sample height. Less than 60 s was needed to inactivate *E. coli* O157:H7 by more than 5‐log CFU/ml at point 4. However, more treatment time was needed to inactivate the pathogen at points 1, 2, and 3. For *E. coli* O157:H7 located at points 1 and 2, 65‐s treatment time was needed to achieve 5‐log reduction, and even longer time was needed to ensure 5‐log reduction at the bottom corner (point 3). In particular, more time was required to ensure 5‐log reduction of pathogens in the largest size sample because the temperature of the coldest point was relatively lower than in smaller samples. The treatment times required to inactivate pathogens by 5‐log CFU/ml at the bottom corner (point 3) were 106, 112, and 118 s for 1, 3, and 5 cm models, respectively. Through the simulation, the exact treatment times to achieve 5 log reduction of *E. coli* O157:H7 were found to follow the size of the chamber.

## 4 CONCLUSION

Orange juice processing by ohmic heating was successfully simulated over time by a numerical simulation approach including fluid dynamics, heat transfer, and pathogen inactivation phenomenon. Because traditional experimental methods usually use survival averages to assess pathogen inactivation, they cannot exactly identify local foodborne pathogens able to survive in corner areas inside the chamber. A united simulation method embedded with our own pathogen inactivation codes using Java language was executed for 1, 3, and 5 cm models. Orange juice temperatures and populations of *E. coli* O157:H7 obtained from the simulation were verified in the 1 cm model. Subsequently, various models with 1, 3, and 5 cm sample heights were simulated following a two‐step processing approach (ohmic heating, and then latent heating) to model factory conditions. During ohmic heating, temperatures increased with treatment time, and concentrations of pathogens decreased accordingly. Rapid convection occurred during the heating time because heated orange juice rose rapidly due to buoyancy. Temperatures at the bottom corner increased most slowly, and accordingly, *E. coli* O157:H7 survived longer there than at the other loci. Moreover, heating nonuniformity was more significant in the larger model than in the smaller model. Consequently, 106, 112, and 118 s were needed to achieve 5‐log reductions of *E. coli* O157:H7 located in the bottom corner in the 1, 3, and 5 cm models, respectively. From these results, we conclude that the bottom corner of the rectangular ohmic heating chamber can be a critical control point (CCP) for inactivating *E. coli* O157:H7 and could pose even more of a food safety hazard when processing larger samples. Therefore, further study simulating the continuous ohmic heater with rounded entry and exit is needed.

## CONFLICT OF INTEREST

The authors declare that they have no competing interest.

## ETHICAL STATEMENTS

This study does not involve any human or animal testing.

## NOMENCLATURE

**Table:** 

Symbol	Value	Parameter	Unit	Note
$V_{0}$	51.2	Applied voltage	V	Initial condition
$c_{0}$	10^5^	Cell concentration	CFU/ml	Initial condition
K	Equation (5)	Thermal conductivity	W/m·K	Singh and Heldman (2001)
$C_{p}$	Equation (6)	Specific heat	J/kg∙K	Singh and Heldman (2001)
ρ	Equation (7)	Density	kg/m^3^	Singh and Heldman (2001)
μ	Equation (8)	Dynamic viscosity	pa∙s	Ibarz et al. (1994)
D	Equation (10)	Thermal diffusivity	m^2^/s	Wilhelm et al. (2005)
σ	Equation (11)	Electrical conductivity	S/m	Palaniappan and Sastry (1991)
$l_{\mathrm{mix}}^{\lim}$	0.07	Admissible mixing length	m	Initial condition
$C_{\varepsilon 1}$	1.44	Model constant	—	—
$C_{\varepsilon 2}$	1.92	Model constant	—	—
$C_{\mu }$	0.09	Model constant	—	—
$\sigma_{k}$	1.0	Model constant	—	—
$\sigma_{\varepsilon }$	1.3	Model constant	—	—
$k_{v}$	0.41	Model constant	—	—

## References

[fsn31353-bib-0001] Ait‐Ouazzou, A. , Espina, L. , García‐Gonzalo, D. , & Pagán, R. (2013). Synergistic combination of physical treatments and carvacrol for *Escherichia coli* O157: H7 inactivation in apple, mango, orange, and tomato juices. Food Control, 32(1), 159–167. 10.1016/j.foodcont.2012.11.036

[fsn31353-bib-0002] Baysal, A. H. , & İçier, F. (2010). Inactivation kinetics of *Alicyclobacillus acidoterrestris* spores in orange juice by ohmic heating: Effects of voltage gradient and temperature on inactivation. Journal of Food Protection, 73(2), 299–304. 10.4315/0362-028X-73.2.299 20132675

[fsn31353-bib-0003] Besser, R. E. , Lett, S. M. , Weber, J. T. , Doyle, M. P. , Barrett, T. J. , Wells, J. G. , & Griffin, P. M. (1993). An outbreak of diarrhea and hemolytic uremic syndrome from *Escherichia coli* O157: H7 in fresh‐pressed apple cider. JAMA, 269(17), 2217–2220. 10.1001/jama.1993.03500170047032 8474200

[fsn31353-bib-0004] Castillo, A. , Villarruel‐López, A. , Navarro‐Hidalgo, V. , Martínez‐González, N. , & Torres‐Vitela, M. (2006). *Salmonella* and *Shigella* in freshly squeezed orange juice, fresh oranges, and wiping cloths collected from public markets and street booths in Guadalajara, Mexico: Incidence and comparison of analytical routes. Journal of Food Protection, 69(11), 2595–2599. 10.4315/0362-028X-69.11.2595 17133801

[fsn31353-bib-0005] Choi, W. , Nguyen, L. T. , Lee, S. H. , & Jun, S. (2011). A microwave and ohmic combination heater for uniform heating of liquid–particle food mixtures. Journal of Food Science, 76(9), E576–E585. 10.1111/j.1750-3841.2011.02413.x 22416703

[fsn31353-bib-0006] Cody, S. H. , Glynn, M. K. , Farrar, J. A. , Cairns, K. L. , Griffin, P. M. , Kobayashi, J. , … Vugia, D. J. (1999). An outbreak of *Escherichia coli* O157: H7 infection from unpasteurized commercial apple juice. Annals of Internal Medicine, 130(3), 202–209. 10.7326/0003-4819-130-3-199902020-00005 10049198

[fsn31353-bib-0007] Cook, K. A. , Dobbs, T. E. , Hlady, W. G. , Wells, J. G. , Barrett, T. J. , Puhr, N. D. , … Swerdlow, D. L. (1998). Outbreak of *Salmonella* serotype Hartford infections associated with unpasteurized orange juice. JAMA, 280(17), 1504–1509. 10.1001/jama.280.17.1504 9809731

[fsn31353-bib-0008] Enache, E. , & Chen, Y. (2007). Survival of *Escherichia coli* O157: H7, *Salmonella*, and *Listeria monocytogenes* in cranberry juice concentrates at different Brix levels. Journal of Food Protection, 70(9), 2072–2077. 10.4315/0362-028X-70.9.2072 17900084

[fsn31353-bib-0009] Fu, W. R. , & Hsieh, C. C. (1999). Simulation and verification of two‐dimensional ohmic heating in static system. Journal of Food Science, 64(6), 946–949. 10.1111/j.1365-2621.1999.tb12257.x

[fsn31353-bib-0010] Ibarz, A. , Gonzalez, C. , & Esplugas, S. (1994). Rheology of clarified fruit juices. III: Orange juices. Journal of Food Engineering, 21(4), 485–494. 10.1016/0260-8774(94)90068-x

[fsn31353-bib-0011] İçi, F. , Yildiz, H. , & Baysal, T. (2008). Polyphenoloxidase deactivation kinetics during ohmic heating of grape juice. Journal of Food Engineering, 85(3), 410–417. 10.1016/j.jfoodeng.2007.08.002

[fsn31353-bib-0012] Ito, R. , Fukuoka, M. , & Hamada-Sato, N. (2014). Innovative food processing technology using ohmic heating and aseptic packaging for meat. Meat Science, 96(2), 675–681. 24200557 10.1016/j.meatsci.2013.10.012

[fsn31353-bib-0013] Kim, S.‐S. , & Kang, D.‐H. (2015). Comparison of pH effects on ohmic heating and conventional heating for inactivation of *Escherichia coli* O157:H7, *Salmonella enterica* Serovar Typhimurium and *Listeria monocytogenes* in orange juice. LWT‐Food Science and Technology, 64(2), 860–866. 10.1016/j.lwt.2015.06.056

[fsn31353-bib-0014] Knoerzer, K. , Regier, M. , & Schubert, H. (2006). Microwave heating: A new approach of simulation and validation. Chemical Engineering & Technology: Industrial Chemistry‐Plant Equipment‐Process Engineering‐Biotechnology, 29(7), 796–801. 10.1002/ceat.200600038

[fsn31353-bib-0015] Lee, S. Y. , Sagong, H. G. , Ryu, S. , & Kang, D. H. (2012). Effect of continuous ohmic heating to inactivate *Escherichia coli* O157: H7, *Salmonella* Typhimurium and *Listeria monocytogenes* in orange juice and tomato juice. Journal of Applied Microbiology, 112(4), 723–731. 10.1111/j.1365-2672.2012.05247.x 22292508

[fsn31353-bib-0016] Leizerson, S. , & Shimoni, E. (2005a). Effect of ultrahigh‐temperature continuous ohmic heating treatment on fresh orange juice. Journal of Agricultural and Food Chemistry, 53(9), 3519–3524. 10.1021/jf0481204 15853396

[fsn31353-bib-0017] Leizerson, S. , & Shimoni, E. (2005b). Stability and sensory shelf life of orange juice pasteurized by continuous ohmic heating. Journal of Agricultural and Food Chemistry, 53(10), 4012–4018. 10.1021/jf047857q 15884832

[fsn31353-bib-0018] Marra, F. (2014). Mathematical model of solid food pasteurization by Ohmic heating: Influence of process parameters. The Scientific World Journal, 2014, 1–8. 10.1155/2014/236437 PMC3914513 24574874

[fsn31353-bib-0019] Oyarzabal, O. A. , Nogueira, M. C. , & Gombas, D. E. (2003). Survival of *Escherichia coli* O157: H7, *Listeria monocytogenes*, and *Salmonella* in juice concentrates. Journal of Food Protection, 66(9), 1595–1598. 10.4315/0362-028X-66.9.1595 14503711

[fsn31353-bib-0020] Palaniappan, S. , & Sastry, S. K. (1991). Electrical conductivities of selected solid foods during ohmic heating 1. Journal of Food Process Engineering, 14(3), 221–236. 10.1111/j.1745-4530.1991.tb00093.x

[fsn31353-bib-0021] Radke, S. M. , & Alocilja, E. C. (2005). A high density microelectrode array biosensor for detection of *E. coli* O157: H7. Biosensors and Bioelectronics, 20(8), 1662–1667. 10.1016/j.bios.2004.07.021 15626625

[fsn31353-bib-0022] Rincón‐Casado, A. , Sánchez de la Flor, F. , Chacón Vera, E. , & Sánchez Ramos, J. (2017). New natural convection heat transfer correlations in enclosures for building performance simulation. Engineering Applications of Computational Fluid Mechanics, 11(1), 340–356. 10.1080/19942060.2017.1300107

[fsn31353-bib-0023] Sarang, S. , Sastry, S. , Gaines, J. , Yang, T. , & Dunne, P. (2007). Product formulation for ohmic heating: Blanching as a pretreatment method to improve uniformity in heating of solid–liquid food mixtures. Journal of Food Science, 72(5), E227–E234. 10.1111/j.1750-3841.2007.00380.x 17995720

[fsn31353-bib-0024] Shim, J. , Lee, S. H. , & Jun, S. (2010). Modeling of ohmic heating patterns of multiphase food products using computational fluid dynamics codes. Journal of Food Engineering, 99(2), 136–141. 10.1016/j.jfoodeng.2010.02.009

[fsn31353-bib-0025] Silva, F. V. , Tan, E. K. , & Farid, M. (2012). Bacterial spore inactivation at 45–65°C using high pressure processing: Study of *Alicyclobacillus acidoterrestris* in orange juice. Food Microbiology, 32(1), 206–211. 10.1016/j.fm.2012.04.019 22850395

[fsn31353-bib-0026] Singh, R. P. , & Heldman, D. R. (2001). Introduction to food engineering. Gulf: Professional Publishing.

[fsn31353-bib-0027] Sospedra, I. , Rubert, J. , Soriano, J. , & Manes, J. (2012). Incidence of microorganisms from fresh orange juice processed by squeezing machines. Food Control, 23(1), 282–285. 10.1016/j.foodcont.2011.06.025

[fsn31353-bib-0028] Timmermans, R. , Groot, M. N. , Nederhoff, A. , Van Boekel, M. , Matser, A. , & Mastwijk, H. (2014). Pulsed electric field processing of different fruit juices: Impact of pH and temperature on inactivation of spoilage and pathogenic micro‐organisms. International Journal of Food Microbiology, 173, 105–111. 10.1016/j.ijfoodmicro.2013.12.022 24418831

[fsn31353-bib-0029] U. S. Food and Drug Administration (U. S. FDA) . (2001). Guidance for Industry: The Juice HACCP Regulation – Questions & Answers. Available from https://www.fda.gov/Food/GuidanceRegulation/GuidanceDocumentsRegulatoryInformation/Juice/ucm072981.htm#F (Accessed 26 October 2018).

[fsn31353-bib-0030] Vikram, V. , Ramesh, M. , & Prapulla, S. (2005). Thermal degradation kinetics of nutrients in orange juice heated by electromagnetic and conventional methods. Journal of Food Engineering, 69(1), 31–40. 10.1016/j.jfoodeng.2004.07.013

[fsn31353-bib-0031] Wilhelm, L. R. , Suter, D. A. , & Brusewitz, G. H. (2005). Physical Properties of Food Materials.St. Joseph, MI: American Society of Agricultural Engineers.

[fsn31353-bib-0032] Ye, X. , Ruan, R. , Chen, P. , & Doona, C. (2004). Simulation and verification of ohmic heating in static heater using MRI temperature mapping. LWT‐Food Science and Technology, 37(1), 49–58. 10.1016/S0023-6438(03)00133-6

